# Higher Serum Bilirubin Levels in Response to Higher Carbohydrate Intake During Early Pregnancy and Lower Gestational Diabetes Mellitus Occurrence in Overweight and Obese Gravidae

**DOI:** 10.3389/fnut.2021.701422

**Published:** 2021-08-30

**Authors:** Wennan He, Liping Wang, Yi Zhang, Yuan Jiang, Xiaotian Chen, Yin Wang, Yalan Dou, Hongyan Chen, Weili Yan

**Affiliations:** ^1^Department of Clinical Epidemiology & Clinical Trial Unit (CTU), Children's Hospital of Fudan University, National Children's Medical Center, Shanghai, China; ^2^International Peace Maternity and Child Health Hospital of China Welfare Institute, Shanghai, China

**Keywords:** carbohydrate, bilirubin, gestational diabetes mellitus, glycemic load, pregnant women

## Abstract

**Background and Aim:** Serum bilirubin levels are recently shown to be a novel protector of gestational diabetes mellitus (GDM), yet whether they could be affected by carbohydrate quality is unclear. We aimed to examine the associations between dietary carbohydrate parameters and serum bilirubin levels during early pregnancy, with further exploration on a potential mediating role of serum bilirubin levels on carbohydrate parameters-GDM pathways.

**Methods:** 260 healthy but overweight or obese gravidae (BMI ≥24 kg/m^2^) derived from a historical cohort in two hospitals in China were included. The associations between carbohydrate parameters (total carbohydrate intake, glycemic index GI, fiber intake, glycemic load GL) and serum bilirubin levels (total bilirubin, TB and direct bilirubin, DB) and GDM were evaluated by multivariable regression analysis. Generalized structural equation modeling was then applied to perform adjusted mediation analysis.

**Results:** Increased serum bilirubin levels (mmol/L) and decreased GDM occurrence were observed following dietary carbohydrate intake (%E) and GL (g/1,000 kcal) in highest tertile compared to the lowest tertile [carbohydrate: TB: β = 0.926 (95%CI: 0.069, 1.782), DB: β = 0.554 (95%CI: 0.192, 0.916);GL:TB: β = 1.170 (95%CI: 0.339, 2.001); DB: β = 0.369 (95%CI: 0.016, 0.700); carbohydrate: adjusted OR = 0.43 (95%CI:0.19–0.99); GL: adjusted OR = 0.36 (95%CI:0.16, 0.84)]. The mediating effect of carbohydrate intake and GL on GDM through bilirubin levels was evaluated as modest (carbohydrate: 6.2% for TB, 1.3% for DB; GL: 8.7% for TB, 2.3% for DB). No association was observed regarding GI and fiber.

**Conclusions:** Mildly elevated serum bilirubin levels appeared to be in response to higher energies consumed from carbohydrate during early pregnancy in healthy overweight or obese gravidae. However, the mediating effect of bilirubin levels on carbohydrate-GDM pathways is not evident. Larger investigation is further needed for solid evidence.

## Introduction

Parallel to the rise in obesity and maternal age, the prevalence of gestational diabetes mellitus (GDM) is increasing worldwide, affecting up to 18% pregnancies globally and approximately 10% in China during last decade (1–3). Since early pregnancy, the alterations in maternal metabolism facilitate peripheral insulin resistance in gravidae, especially those with high pre-pregnancy BMI, and thereby at a higher risk of GDM (4). Therefore, identifying early indicators in GDM pathogenesis should be beneficial in disease prevention in this at-risk population.

Previous experimental evidence supported a protective role of heme catabolic pathways in pathogenesis of diabetes mellitus (5). Bilirubin, as the end product of heme catabolism in the systemic circulation, were recently found to have salutary effects on prevention of diabetes mellitus (6) through anti-oxidative cytoprotective properties, antioxidant actions, and anti-inflammatory effects (5). Mildly elevated serum bilirubin has been demonstrated to inhibit lipoprotein oxidation and up-regulation of several pro-inflammatory biomarkers such as endothelial adhesion molecules and c-reactive protein (5, 7–9). A prospective cohort study of nearly 3,000 gravidae reported that women with higher serum direct bilirubin levels during mid- pregnancy have lower GDM risk (10).

According to several reviews, chronic inflammation and oxidation underlies the pathogenesis of insulin resistance, which could be altered by dietary macronutrients, and dietary carbohydrate quality is one of them (11–13). Yet, as a novel protector of GDM, whether serum bilirubin levels in response to carbohydrate parameters is unknown. Since carbohydrate is the cornerstone in GDM diets, it is possible that bilirubin predisposes GDM through similar mechanisms, which could be modified by different carbohydrate quality. However, this has not been explored.

This study was whereby conducted, with the aim to examine if bilirubin could be affected by carbohydrate quality, and further explore if bilirubin is a potential mediator in the carbohydrate quality-GDM pathway. The findings from this study will help provide a novel mechanistic insight into the pathogenesis of GDM influenced by carbohydrate parameters and bilirubin. In addition, the results will be helpful in identifying early indicator of GDM, especially for dietary modification in overweight and obese gravidae, who are greatly predisposed to GDM.

## Materials and Methods

### The Cohort

This study was a secondary data analysis using a historical cohort (ClinicalTrials.gov registry: NCT01628835). Details of the previous study design, recruitment, methods and responses could be found elsewhere (14). Briefly, overweight or obese gravidae [BMI ≥24 kg/m^2^ according to national consensus of BMI cut-off for Chinese adults (15)] aged 20–45 years old were recruited at first antenatal visit ≤ 14 gestational weeks in two hospitals [Kunshan Maternity and Child Care Center (Kunshan city, Jiangsu province, China) and the International Peace Maternity and Child Health Hospital of China Welfare Institute (Shanghai, China)] from June 2012 to October 2015. After excluding those with multiple pregnancy, artificial impregnation, special diets (e.g., vegans) and diagnosed chronic diseases (a history of hypertension, diabetes, heart diseases, mental disorders), 400 gravidae were eligible for follow-up till delivery. In the previous research, participants were randomly allocated to receive either standard (*n* = 200) or individualized dietary counseling (*n* = 200) throughout the pregnancy. The researchers found two counseling approaches received similar effectiveness on maternal and neonatal outcomes (including GDM, macronutrient intakes, and insulin or glucose levels). All participants provided written informed consent before baseline data collection.

### Dietary Assessment

Dietary intake was assessed by 24-h dietary recall. Trained clinical dieticians asked participants to report their food intake in one recent weekday before the visit, which best reflected their regular dietary habit. Portion-size food molds were used to assess the serving sizes. Because carbohydrate quality was the principal consideration when matching a particular food with one in food lists, nutrient intakes were calculated primarily based on Chinese nutrient composition tables with published GI values of over 200 food items (16, 17). If the food was not available in the Chinese food lists, the food assignment was further updated to incorporate newly published GI values from international standards (18–20). Daily intakes of total carbohydrate (g) and fiber (g) were whereby calculated. Dietary GI was averaged during daily meals. The value of carbohydrate intake (g) of each consumed food was then multiplied by the respective GI to obtain the daily glycemic load (GL) (18).

The carbohydrate parameters (total carbohydrate intake, fiber intake, dietary GI, GL) were converted to relative intakes adjusted by total energy intake using residual nutrient density models (21). Total carbohydrate intake was computed as percentage of energy (%E) by using the following equation: *Total carbohydrate intake*(*%E*) = (*total carbohydrate intake* (*g*) × 4) ÷ *total energy intake* (*kcal*) × 100%. Fiber intake and GL were computed as grams for every 1,000 kcal. Validation of relative intakes among dietary parameters was assessed by intercorrelations (see Supplementary Table 1). We also grouped participants into tertiles by intakes of total carbohydrate, fiber, and GL. Dietary GI was grouped into three levels (low GI: ≤ 55, medium GI: 55–70, high GI: ≥70) according to international consensus (22).

### Non-Dietary Assessment

At first antenatal visit, demographic characteristics and gestational lifestyle factors were collected through self-reported questionnaire in all participants. Anthropometric measurements and blood samples were collected at the same visit. BMI was calculated as weight (kg)/height (m)^2^. The gestational week was estimated from self-reported last menstrual period and corrected by the first routine ultrasound examinations. GDM was defined based on the routine 75 g oral glucose tolerance test (OGTT) screening mostly at 24 gestation weeks (few deferred but not later than 28 gestational weeks) according to international standards (23, 24). Baseline laboratory data was directly extracted from the hospital information system, which included serum bilirubin, fasting plasma glucose, serum insulin, lipid profiles, glycosylated hemoglobin (HbA1c), and blood pressure. The homeostasis model assessment of insulin resistance (HOMA-IR) was calculated as follows: [fasting plasma glucose (mmol/L)] × (fasting insulin [mIU/L])/22.5 (25). Gestational weight gain was calculated as body weight (kg) difference between baseline and antenatal visit at 24 gestational weeks.

### Participants Included in This Secondary Study

In this secondary study, we reused the pre-existing data of the same cohort. The baseline dietary data and OGTT results were available in all 400 subjects. We further excluded those without bilirubin measurements (*n* = 130), implausible dietary intake in regards to total energy intake (<500 or >3,500 kcal/day) and prescribed medication use since 3 months before pregnancy. As a result, a total of 260 participants were included.

### Statistical Analysis

Characteristics of study participants were expressed as the mean (standard deviation) for normally distributed variables, median (inter-quartile range) for skewed variables and frequency with percentage for categorical variables. Group comparisons were performed by Bonferroni multiple-comparison test, Kruskal-Wallis test and χ^2^-tests, respectively.

**Figure 1:**
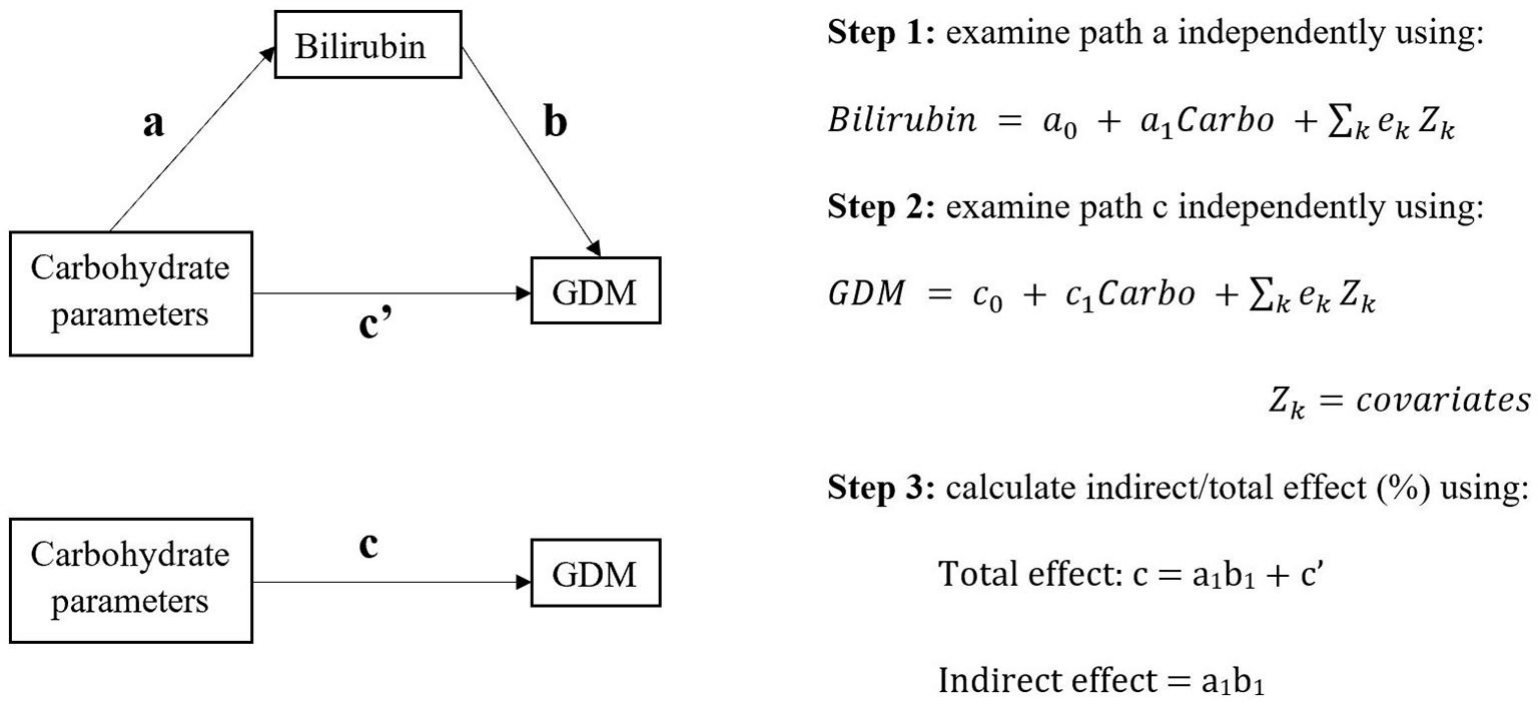
The study design of potential causal pathways among carbohydrate parameters, bilirubin, and gestational diabetes mellitus (GDM).

We firstly examined two paths (path a: carbohydrate parameter-bilirubin; path c: carbohydrate parameter-GDM, see Figure 1) separately by multivariable regression analysis, which were in line with the first two conditions in Baron & Kenny's four-step sequential verification models (26, 27). Carbohydrate parameters were treated as continuous or in groups. Bilirubin were serum total bilirubin (TB) and direct bilirubin (DB) levels (mmol/L).

Covariates were selected as a priori based on previous literatures (2, 5, 28–31) and the authors' background knowledge, which included gestational week, maternal age (years), total energy intake (kcal), pregnancy alcohol drinking (yes, no), baseline BMI (kg/m^2^), baseline fasting plasma glucose (mmol/L), and triglycerides (mmol/L). Cohort-specific covariates (hospital and dietary counseling) were also included. Total carbohydrate intake, fiber intake, and dietary GI were adjusted from the other two in order to minimize interference among carbohydrate parameters. Pregnancy smoking, maternal education, and household income were not included in the analysis due to highly missing values (>50%). Physical activity was not available for analysis because of qualitative design.

A potential association was defined as 95% confidence interval of estimates beyond null after adjusting for the abovementioned covariates. If the two paths were consistent for the same carbohydrate parameter, generalized structural equation modeling (GSEM) would be then applied to determine total and indirect effect of carbohydrate parameter on GDM through bilirubin. The GSEM model constructed three paths (exposure, mediator, and outcome) simultaneously with adjustment of same covariates as before. GSEM calculated total and indirect effect based on Baron and Kenny's mediation model [(26); Figure 1]. The proportion of mediating effect was calculated as indirect effect/total effect × 100%.

To examine the robustness of the observed associations, we also compared baseline metabolic status of mothers (see Supplementary Table 2) and further adjusted for other well-known risk factors of GDM (2, 28, 29, 31) in sensitivity analysis. This included parity (0, 1, ≥2), folic acid and vitamin supplement since 3 months pre-pregnancy till baseline (folic acid + vitamin, folic acid only, vitamin only, no, or unknown), family history of diabetes or hypertension (yes, no), gestational weight gain (kg), and gestational hypertension (yes, no).

The data analysis was performed with STATA (Version 15.1, StataCorp L.P., and College Station, TX). The significance level for Bonferroni, Kruskal-Wallis, χ^2^-tests and GSEM was set at two-sided *p* < 0.05.

## Results

### Characteristics of Study Population

Characteristics of study population are summarized in Table 1. At baseline, the participants reported average intakes of 1,485 (±471) kcal total energy, with 54.2 (±12.7) %E total carbohydrate and 88.4 (±26.2) g/1,000 kcal GL. Fiber intake was 6.2 g/1,000 kcal (25th:4.7, 75th:8.4). Daily GI was 66.1 (25th:60.6, 75th:70.3). Baseline serum TB was 9.4 (±2.9) mmol/L. Baseline serum DB was 3.1 (±1.2) mmol/L. 57 (21.9%) participants developed GDM.

**Table 1:** Baseline characteristics of all study participants^a^.

	***N*** **= 260**
**Age (year)**	28.8 (3.3)
**Gestational week (week)**	12.3 (1.7)
**BMI **(kg/m**^**2**^)**	28.5 (3.0)
**Gestational weight gain** ^**b**^ **(kg)**	5.8 (4.0)
**Hospital**	
Kunshan	43 (16.5%)
International Peace	217 (83.5%)
**Dietary counseling**	
Standard	130 (50.0%)
Individualized	130 (50.0%)
**Total energy intake (kcal)**	1,485 (471)
**Total carbohydrate intake (%E)**	54.2 (12.7)
**Fiber intake** ^**c**^ **(g/1,000 kcal)**	6.2 (4.7,8.4)
**Dietary glycemic index** ^**c**^	66.1 (60.6,70.3)
**Dietary glycemic load (g/1,000 kcal)**	88.4 (26.2)
**Pregnancy alcohol drinking**	3 (1.2%)
**Gestational hypertension**	53 (20.6%)
**Parity**	
0	91 (35.0%)
1	32 (12.3%)
≥2	19 (7.3%)
Unknown	118 (45.4%)
**Family history of diabetes or hypertension**	
Yes	103 (39.6%)
No	94 (36.2%)
Unknown	63 (24.2%)
**Folic acid and vitamin supplement** ^**d**^	
Unknown or no	22 (8.5%)
Vitamin only	36 (13.9%)
Folic acid only	39 (15.0%)
Vitamin + folic acid	163 (62.7%)
**Fasting plasma glucose (mmol/L)**	4.7 (0.4)
**TG** ^**c**^ **(mmol/L)**	1.5 (1.1, 2.0)
**TB (mmol/L)**	9.4 (2.9)
**DB (mmol/L)**	3.1 (1.2)
**GDM**	57 (21.9%)

a *The distribution was summarized as mean (standard deviation) or frequency (%) if not specified*.

b *From baseline till 24 gestational weeks*.

c *Median (25th,75th)*.

d *Since 3 months pre-pregnancy till baseline*.

*BMI, body mass index; TG, triglyceride; %E, percentage of total energy; w, week; y, years; BMI, body mass index; TB, total bilirubin; DB, direct bilirubin; GDM, gestational diabetes mellitus*.

When carbohydrate parameters were grouped in Table 2, most maternal characteristics except gestational hypertension were generally balanced across different levels of carbohydrate parameters. Lower bilirubin levels were observed in those having lower GI foods compared to high GI foods. We also found that both serum bilirubin levels increased as carbohydrate intake (%E) increased by tertiles.

**Table 2:** Baseline characteristics of study population by carbohydrate parameters^a^.

	**Total carbohydrate intake (%E)**			***P***	**Fiber intake (g/1,000 kcal)**			***P***
	**T1** **(***n*** = 87)**	**T2** **(***n*** = 87)**	**T3** **(***n*** = 86)**		**T1** **(***n*** = 87)**	**T2** **(***n*** = 87)**	**T3** **(***n*** = 86)**	
Age (y)	29.3 (3.4)	28.7 (3.0)	28.4 (3.3)	0.19	28.8 (3.3)	29.1 (3.3)	28.5 (3.3)	0.49
Gestational week (w)	12.4 (1.6)	12.3 (1.8)	12.1 (1.6)	0.50	12.5 (1.6)	12.2 (1.3)	12.2 (2.1)	0.48
BMI (kg/m^2^)	28.9 (3.3)	28.0 (2.8)	28.4 (2.9)	0.10	28.9 (3.3)	28.6 (3.0)	28.0 (2.7)	0.11
Gestational weight gain^b^ (kg)	6.0 (3.3)	5.8 (4.4)	5.7 (4.2)	0.88	5.8 (3.6)	5.8 (4.1)	5.9 (4.3)	0.96
Total energy intake (kcal)	1,420 (492)	1,587 (487)	1,447 (419)	0.04^*^	1,525 (470)	1,449 (480)	1,481 (466)	0.56
Pregnancy alcohol drinking^c^	2 (2.3%)	0	1 (1.1%)	0.36	0	3 (3.5%)	0	0.04^*^
Gestational hypertension	27 (31.0%)	16 (18.4%)	10 (11.6%)	0.01^*^	27 (31.0%)	10 (11.5%)	16 (18.6%)	0.01^*^
**Parity**								
0	26 (29.9%)	35 (40.2%)	30 (34.9%)	0.42	26 (30.0%)	41 (47.1%)	24 (27.9%)	0.03^*^
1	8 (9.2%)	12 (13.8%)	12 (14.0%)		13 (15.0%)	10 (11.5%)	9 (10.5%)	
≥2	5 (5.8%)	6 (6.9%)	8 (9.3%)		5 (5.8%)	3 (3.5%)	11 (12.8%)	
Unknown	48 (55.2%)	34 (39.1%)	36 (41.9%)		43 (49.4%)	33 (37.9%)	42 (48.8%)	
**Family history of diabetes or hypertension**								
Yes	33 (37.9%)	34 (39.1%)	36 (41.9%)	0.31	31 (35.6%)	33 (37.9%)	39 (45.4%)	0.35
No	27 (31.0%)	37 (42.5%)	30 (34.9%)		29 (33.3%)	34 (39.1%)	31 (36.0%)	
Unknown	27 (31.1%)	16 (18.4%)	20 (23.2%)		27 (31.0%)	20 (23.0%)	16 (18.6%)	
**Folic acid and vitamin supplement** ^c^								
Unknown or no	8 (9.2%)	6 (6.9%)	8 (9.3%)	0.79	4 (4.6%)	7 (8.1%)	11 (12.8%)	0.55
Vitamin only	11 (12.6%)	11 (12.6%)	14 (16.3%)		15 (17.2%)	10 (11.5%)	11 (12.8%)	
Folic acid only	12 (13.8%)	11 (12.6%)	16 (18.6%)		14 (16.1%)	13 (14.9%)	12 (14.0%)	
Vitamin + folic acid	56 (64.4%)	59 (67.8%)	48 (55.8%)		54 (62.1%)	57 (65.5%)	52 (60.5%)	
**Hospital**								
Kunshan	9 (10.3%)	13 (14.9%)	21 (24.4%)	0.04^*^	9 (10.3%)	14 (16.1%)	20 (23.3%)	0.07
International Peace	78 (89.7%)	74 (85.1%)	65 (75.6%)		78 (89.7%)	73 (83.9%)	66 (76.7%)	
**Dietary counseling**								
Standard	48 (55.2%)	37 (42.5%)	45 (52.3%)	0.21	43 (49.4%)	40 (46.0%)	47 (54.7%)	0.51
Individualized	39 (44.8%)	50 (57.5%)	41 (47.7%)		44 (50.6%)	47 (54.0%)	39 (45.4%)	
Fasting plasma glucose (mmol/L)	4.7 (0.4)	4.7 (0.5)	4.7 (0.4)	0.90	4.6 (0.4)	4.7 (0.5)	4.7 (0.4)	0.35
TG (mmol/L)	1.6 (1.2, 2.1)	1.4 (1.1, 1.8)	1.4 (1.1, 2.1)	0.22	1.6 (1.2, 2.1)	1.4 (1.1, 1.9)	1.4 (1.1, 1.9)	0.09
TB (mmol/L)	8.5 (2.4)	9.7 (2.9)	9.9 (3.1)	0.002^*^	9.2 (2.9)	9.6 (3.1)	9.3 (2.6)	0.73
DB (mmol/L)	2.8 (1.0)	3.1 (1.2)	3.3 (1.3)	0.02^*^	3.2 (1.2)	3.1 (1.2)	3.1 (1.3)	0.85
GDM	25 (28.7%)	20 (23.0%)	12 (14.0%)	0.06	22 (25.3%)	15 (17.2%)	20 (23.3%)	0.41
	**Dietary glycemic index**			***P***	**Dietary glycemic load (g/1,000 kcal)**			***P***
	**Low** **(***n*** =** **26)**	**Medium** **(***n*** = 167)**	**High** **(***n*** = 67)**		**T1** **(***n*** = 87)**	**T2** **(***n*** = 87)**	**T3** **(***n*** = 86)**	
Age (y)	28.5 (1.8)	28.9 (3.5)	28.6 (3.0)	0.70	29.1 (3.5)	28.8 (2.9)	28.6 (3.5)	0.61
Gestational week (w)	12.0 (1.2)	12.2 (1.7)	12.5 (1.8)	0.32	12.2 (1.5)	12.5 (1.9)	12.2 (1.6)	0.27
BMI (kg/m^2^)	27.8 (3.1)	28.3 (2.8)	29.2 (3.5)	0.05	28.8 (3.4)	28.1 (2.6)	28.5 (2.9)	0.27
Gestational weight gain^b^ (kg)	7.1 (5.4)	5.9 (4.0)	5.3 (3.3)	0.19	5.8 (3.)	6.3 (5.0)	5.4 (3.5)	0.43
Total energy intake (kcal)	1,615 (486)	1,450 (484)	1,523 (429)	0.19	1,465 (481)	1,513 (475)	1,478 (463)	0.78
Pregnancy alcohol drinking^c^	0	2 (1.2%)	1 (1.5%)	0.83	1 (1.2%)	1 (1.2%)	1 (1.2%)	1.00
Gestational hypertension	5 (19.3%)	34 (20.36%)	14 (20.9%)	0.65	26 (19.9%)	14 (16.1%)	13 (15.1%)	0.04^*^
**Parity**								
0	5 (19.2%)	57 (34.1%)	29 (43.3%)	0.08	24 (17.6%)	34 (39.1%)	33 (38.4%)	0.19
1	5 (19.2%)	18 (10.8%)	9 (13.4%)		11 (12.6%)	9 (10.3%)	12 (13.4%)	
≥2	1 (3.9%)	17 (10.2%)	1 (1.49%)		4 (4.6%)	10 (11.5%)	5 (5.9%)	
Unknown	15 (57.7%)	75 (44.9%)	28 (41.8%)		48 (55.17%)	34 (39.1%)	36 (41.9%)	
**Family history of diabetes or hypertension**								
Yes	9 (34.6%)	72 (43.1%)	22 (32.8%)	0.55	31 (35.6%)	35 (40.2%)	37 (43.0%)	0.52
No	10 (38.5%)	55 (32.9%)	29 (43.3%)		30 (34.5%)	35 (40.2%)	29 (33.7%)	
Unknown	7 (26.9%)	40 (24.0%)	16 (23.9%)		26 (29.9%)	17 (19.6%)	20 (23.3%)	
**Folic acid and vitamin supplement** ^c^								
Unknown or no	4 (15.4%)	12 (7.2%)	6 (9.0%)	0.55	9 (10.3%)	7 (8.1%)	6 (7.0%)	0.86
Vitamin only	6 (23.1%)	21 (12.6%)	9 (13.4%)		13 (14.9%)	9 (10.3%)	14 (16.3%)	
Folic acid only	4 (15.4%)	25 (15.0%)	10 (14.9%)		11 (12.6%)	15 (17.2%)	13 (15.1%)	
Vitamin + folic acid	12 (46.2%)	109 (65.3%)	42 (62.7%)		54 (62.1%)	56 (64.4%)	53 (61.6%)	
**Hospital**								
Kunshan	4 (15.4%)	30 (18.0%)	9 (13.4%)	0.69	11 (12.6%)	15 (17.2%)	17 (19.8%)	0.44
International Peace	22 (84.6%)	137 (82.0%)	58 (86.6%)		76 (87.4%)	72 (82.8%)	69 (80.2%)	
**Dietary counseling**								
Standard	13 (50.0%)	83 (49.7%)	34 (50.8%)	0.99	44 (50.6%)	42 (48.3%)	44 (51.2%)	0.92
Individualized	13 (50.0%)	84 (50.3%)	33 (49.2%)		43 (49.4%)	45 (51.7%)	42 (48.8%)	
Fasting plasma glucose (mmol/L)	4.5 (0.4)	4.7 (0.4)	4.7 (0.5)	0.06	4.6 (0.4)	4.7 (0.4)	4.7 (0.5)	0.62
TG (mmol/L)	1.2 (1.1, 1.6)	1.5 (1.2, 2.0)	1.6 (1.2, 2.1)	0.15	1.5 (1.1, 2.2)	1.4 (1.2, 1.9)	1.4 (1.1, 2.1)	0.96
TB (mmol/L)	9.4 (2.6)	9.0 (2.8)	10.2 (3.1)	0.01^*^	8.6 (2.4)	9.5 (2.9)	10.1 (3.1)	0.003^*^
DB (mmol/L)	3.24 (1.2)	3.0 (1.2)	3.4 (1.2)	0.02^*^	2.9 (1.1)	3.1 (1.3)	3.3 (1.2)	0.06
GDM	3 (11.5%)	41 (24.6%)	13 (19.4%)	0.27	25 (28.7%)	21 (24.1%)	11 (12.8%)	0.03^*^

a *Continuous values are presented as median (25th,75th) for TG or mean (standard deviation) otherwise*.

b *Since baseline till 24 gestational weeks*.

c *Light alcohol drinkers*.

d *Since 3 months before pregnancy till baseline*.

* *Two-sided P < 0.05 among groups*.

*T, tertile; %E, percentage of total energy; w, week; y, years; BMI, body mass index; TG, triglyceride; TB, total bilirubin; DB, direct bilirubin; GDM, gestational diabetes mellitus*.

### Carbohydrate Parameters and Serum Bilirubin Levels

Serum bilirubin levels were positively associated with total carbohydrate intake (%E) and GL (g/1,000 kcal) (Table 3) at baseline after adjusting for covariates. TB increased 0.030 (95%CI: 0.002, 0.058) mmol/L for every 1%E increase in total carbohydrate while 0.014 (95%CI: 0.002, 0.026) mmol/L elevation for DB correspondingly. The magnitudes of coefficients were amplified when total carbohydrate intake was grouped into tertiles. There were 0.865 mmol/L (95%CI: 0.009, 1.721) and 0.926 mmol/L (95%CI: 0.069, 1.782) increase in TB for tertile 2 and 3, respectively, compared to the lowest tertile. Similarly, DB increased 0.303 (95%CI: −0.058, 0.665) mmol/L in tertile 2 and 0.554 (95%CI: 0.192, 0.916) mmol/L in tertile 3 compared to the lowest tertile. Dietary GL showed similar trends. Bilirubin levels rose as GL increased by 1 g/1,000 kcal [TB: 0.021 (95%CI: 0.008, 0.034); DB: 0.006 (95%CI: 0.001, 0.010)] as well as in the highest GL tertile [TB: 1.170 (95%CI: 0.339, 2.001), DB: 0.369 (95%CI: 0.016, 0.700)] compared to the lowest tertile. Conversely, we did not find the association between dietary GI or fiber with bilirubin levels, based on estimates with wide 95%CI in either direction (Table 3).

**Table 3:** Associations of carbohydrate parameters with serum bilirubin levels^a^.

	**Adjusted regression coefficients (95%CI)**			
	**TB (mmol/L)**	***P***	**DB (mmol/L)**	***P***
	***n*** **= 259**		***n*** **= 260**	
**Total carbohydrate intake**				
Change per 1%E increase in total carbohydrate intake	0.030 (0.002, 0.058)^*^	0.03	0.014 (0.002, 0.026)^*^	0.02
Tertile 1	Reference			
Tertile 2	0.865 (0.009, 1.721)^*^	0.04	0.303 (−0.058, 0.665)	0.10
Tertile 3	**0.926** (0.069, 1.782)^*^	0.03	**0.554** (0.192, 0.916)^*^	0.003
**Fiber intake**				
Change per 1 g/1,000 kcal increase in fiber intake	−0.044 (−0.132, 0.045)	0.33	−0.018 (−0.055, 0.020)	0.35
Tertile 1	Reference			
Tertile 2	0.023 (−0.825, 0.873)	0.95	−0.235 (−0.595, 0.124)	0.19
Tertile 3	−0.165 (−1.049, 0.718)	0.71	−0.110 (−0.484, 0.264)	0.56
**Dietary glycemic index**				
Change per 1 increase in dietary GI	0.024 (−0.014, 0.063)	0.20	−0.004 (−0.020, 0.012)	0.65
Low (≤ 55)	Reference			
Medium (55–70)	−0.339 (−1.493, 0.814)	0.56	−0.342 (−0.834, 0.140)	0.17
High (≥70)	0.747 (−0.566, 2.061)	0.26	−0.029 (−0.588, 0.530)	0.91
**Dietary glycemic load**				
Change per 1 g/1,000 kcal increase in dietary GL	0.021 (0.008, 0.034)^*^	0.002	0.006 (0.001, 0.010)^*^	0.04
Tertile 1	Reference			
Tertile 2	0.631 (−0.206, 1.469)	0.13	0.103 (−0.253, 0.450)	0.57
Tertile 3	**1.170** (0.339, 2.001)^*^	0.006	0.369 (0.016, 0.700)^*^	0.04

a *Adjusted for pregnancy alcohol drinking, baseline BMI, gestational week, maternal age, total energy intake, dietary counseling, hospital, baseline fasting glucose and triglyceride levels. Adjustment within carbohydrate parameters: carbohydrate: fiber and GI, fiber: carbohydrate and GI; GI: carbohydrate and fiber*.

* *Estimates with 95%CI beyond null*.

*%E, percentage of total energy; TB, total bilirubin; DB, direct bilirubin; CI, confidence interval*.

The sensitivity analysis of further adjustment on risk factors of GDM, showed attenuated but consistent associations (see Supplementary Table 3).

### Carbohydrate Parameters and GDM

**Figure 2:**
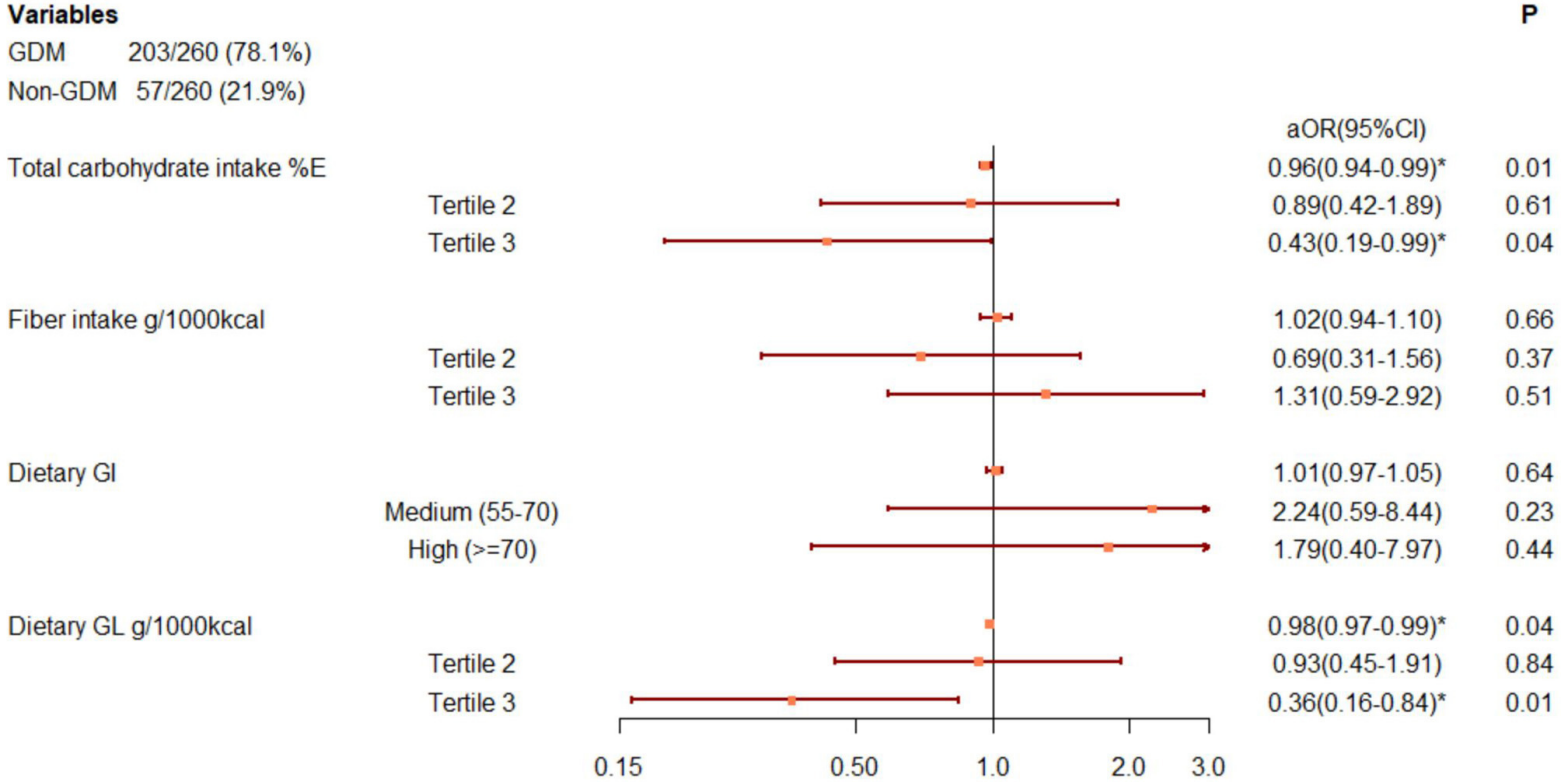
Associations between carbohydrate parameters and GDM onset, after adjusting for pregnancy alcohol drinking, baseline BMI, gestational week, maternal age, total energy intake, dietary counseling, hospital, baseline fasting glucose, and triglyceride levels. Adjustment within carbohydrate parameters: carbohydrate: fiber and GI, fiber: carbohydrate and GI; GI: carbohydrate and fiber. ^*^Adjusted odds ratio with 95%CI beyond null. %E, percentage of total energy, d, day, GI, glycemic index, GL, glycemic load, GDM, gestational diabetes mellitus.

As shown in Figure 2, total carbohydrate intake (%E) and GL (g/1,000 kcal) at baseline was inversely associated with GDM onset. The associations became stronger in the highest tertile compared to the lowest tertile [carbohydrate: adjusted OR: 0.43 (95%CI: 0.19, 0.99); GL: adjusted OR: 0.38 (95%CI: 0.17, 0.86)]. We found no association between fiber intake or GI on later GDM occurrence, either in continuous scale or in categories, given a wide 95%CI containing null value (Figure 2).

The sensitivity analysis of further adjustment on risk factors of GDM showed attenuated but consistent results (see Supplementary Table 4).

### Mediation Analysis

The results of mediation analysis for carbohydrate/GL-TB/DB-GDM pathways were presented in Tables 4, 5. The potential effect of carbohydrate or GL, either in total or indirectly through bilirubin levels, were inversely associated with GDM onset since all estimates were skewed to negative values. Yet, whereas the carbohydrate and GL had significant total effects on GDM, the mediating effects of TB and DB were not significant. The proportions of indirect effects showed that around 6.2% of the effect of carbohydrate intake (%E) on GDM was mediated by TB level, while 1.3% coming from DB level. Nearly 8.7% of the effect of GL (g/1,000 kcal) on GDM was indirectly through TB level and 2.3% through DB level. When carbohydrate and GL was measured by tertiles, the proportion of indirect effect increased for carbohydrate, as 8.3% through TB and 2.0% through DB in the pathways. GL tertiles showed similar proportion of indirect effect as GL in g/1,000 kcal (Table 5).

**Table 4:** Effect of carbohydrate intake and GL on GDM in total and indirectly through bilirubin levels.

**Effects**	**Pathway**	**Coef. (95%CI)**	**SE**	***Z***	***P*** **-value**	**Proportion**
**TB** **(***n*****=** 259)**						
Indirect	Carbohydrate → TB → GDM	−0.00034 (−0.0011, 0.00044)	0.00040	−0.85	0.394	6.2%
Total	Carbohydrate → GDM	−0.0056 (−0.0096, −0.0015)	0.0020	−2.71	0.007	
Indirect	GL → TB → GDM	−0.00023 (−0.00064,0.00027)	0.00023	−0.81	0.418	8.7%
Total	GL → GDM	−0.0021 (−0.0040, −0.00024)	0.00097	−2.21	0.027	
**DB** **(***n*****=** 260)**						
Indirect	Carbohydrate → DB → GDM	−0.00070 (−0.00059, 0.00045)	0.00026	−0.26	0.793	1.3%
Total	Carbohydrate → GDM	−0.0052 (−0.0092, −0.0012)	0.0020	−2.55	0.011	
Indirect	GL → DB → GDM	−0.000045 (−0.00028,0.00019)	0.00012	−0.37	0.713	2.3%
Total	GL → GDM	−0.0020 (−0.0039,−0.00009)	0.00097	−2.06	0.040	

*GDM, gestational diabetes mellitus; Coef. Regression coefficient; CI, confidence interval; SE, standard error; TB, total bilirubin; DB, direct bilirubin*.

**Table 5:** Effect of carbohydrate and GL tertiles on GDM in total and indirectly though bilirubin levels.

**Effects**	**Pathway**	**Coef. (95%CI)**	**SE**	***Z***	***P*** **-value**	**Proportion**
**TB** **(***n*****=** 259)**						
Indirect	Carbohydrate → TB → GDM	−0.0057 (−0.018, 0.0064)	0.0062	−0.92	0.359	8.3%
Total	Carbohydrate → GDM	−0.068 (−0.13, −0.0058)	0.032	−2.14	0.032	
Indirect	GL → TB → GDM	−0.0051 (−0.017,0.0075)	0.0064	−0.79	0.428	6.5%
Total	GL → GDM	−0.078 (−0.138, −0.018)	0.030	−2.55	0.011	
**DB** **(***n*****=** 260)**						
Indirect	Carbohydrate → DB → GDM	−0.0013 (−0.011, 0.0086)	0.0051	−0.26	0.798	2.0%
Total	Carbohydrate → GDM	−0.064 (−0.13, −0.0019)	0.043	−2.02	0.043	
Indirect	GL → DB → GDM	−0.0013 (−0.009, 0.0068)	0.0041	−0.32	0.747	1.9%
Total	GL → GDM	−0.074 (−0.13, −0.015)	0.031	−2.43	0.015	

*GDM, gestational diabetes mellitus; Coef. Regression coefficient; CI, confidence interval; SE, standard error; TB, total bilirubin; DB, direct bilirubin*.

## Discussion

Overall, this study reused a pre-existing data of overweight and obese gravidae, with initial exploration on associations of serum bilirubin levels with carbohydrate quality, and indirect effect of carbohydrate intake (mediated through bilirubin) on GDM. Participants consuming higher total carbohydrate intake (%E) and dietary GL (g/1,000 kcal) in early pregnancy appeared to have higher serum bilirubin levels and were less likely to suffer from GDM. Meanwhile, bilirubin levels were hypothesized as a potential mediator in the association between carbohydrate/GL and GDM. However, the mediation analysis suggested the indirect effect through bilirubin was minimal compared to direct effect.

Carbohydrate-rich diets have been expected to increase diabetes risk by directly facilitating post-prandial glucose concentrations (32). However, mounting evidence supports carbohydrate restriction at the cost of increasing calories from fat intake as less optimal according to recent reviews (33, 34). Although limiting carbohydrate helps control glycemia, substituting fat for carbohydrate in obese women with pre-pregnancy insulin resistance may promote intrauterine overnutrition (33–35). Furthermore, diets high in fat may promote insulin resistance partially through elevation of free fatty acid (FFAs), which concomitantly impaired insulin signaling (36). These underlying mechanisms may explain the direct effect of carbohydrate intake (%E) on GDM. In our data, we observed a high intercorrelation between carbohydrate intake (%E) and fat intake (%E) (*r* = −0.93, Supplementary Table 1). While we were not sure about the reason for the highly negative correlation, the observed associations might be explained by replacement of fat intake, which were in line with previous results from observational studies and clinical trials, that high-carbohydrate with low-fat diets led to decreased risk of GDM and lower fasting glucose in GDM patients (28, 31, 37–40).

Bilirubin has been recognized as a product of heme catabolism with potent antioxidant and anti-inflammatory properties (5). Given a potential link between different carbohydrate parameters with alteration on inflammatory markers and oxidative indicators (41–43), we believe bilirubin can be modified by different carbohydrate quality. In this study, we are the first that found both serum total and direct bilirubin levels increased mildly following higher energies from total carbohydrate intake in overweight and obese gravidae. Although limited by the absence of previous evidence, we consider the directions of the associations as reasonable since replacement of fat (%E) with carbohydrate (%E) has shown potential antioxidative and anti-inflammatory effects (44–47). Despite this, whether bilirubin was directly induced by carbohydrate intake or regulated through other biomarkers or key products in heme catabolism still needs further exploration.

However, mediation analysis by GSEM indicated only marginal non-significant effect of bilirubin levels involving in the carbohydrate-GDM association. This is probably because the total effect of carbohydrate on GDM was already mild. As a consequence, it precluded obvious mediating effect to be detected from statistical models, and further masked if different mediators existed and produced opposite effects. As bilirubin was a potentially negative mediator, its effect could be partially neutralized by other mediators that increased GDM, particularly in longitudinal studies (48). Given that the mediated relationship is an intrinsically causal relationship (26), this observational design only provided conservative estimations that should be interpreted with caution. If the observed mediation was true, the proportions of indirect effect suggested around 8% in the association was contributed by TB level and <3% was responsible for DB level. Because DB was a subset of TB (49), it might explain the difference in magnitude and indicate that the mediating effect was through overall bilirubin metabolism rather than bilirubin subtypes. Further exploration with a wide coverage of biomarkers involved in bilirubin homeostasis or heme catabolism might help provide a bigger picture.

In our study, dietary GL showed similar results as carbohydrate. The observed association might be explained by the intercorrelation that the dietary GL in this study was affected mainly by carbohydrate intake rather than GI (*r*= 0.86 with carbohydrate and 0.61 with GI, Supplementary Table 1). This led to limited implication for GL in terms of its comprehensive effect accounting for both carbohydrate quantity and quality. Even so, we still observed higher proportions of indirect effect of bilirubin levels for GL compared to carbohydrate intake. This gap was narrowed regarding tertiles of carbohydrate and GL. These suggested that bilirubin response on GL-GDM and carbohydrate-GDM pathways was seemingly different. This hypothesis should be investigated in a future study.

In addition, we failed to detect an association regarding either GI or fiber. The discrepancies were possibly due to the high skewness in GI and fiber intake in our study, which impeded comparison between extreme levels and underestimated the associations. Besides, as low-GI foods often have high fiber content (34), the adjustment within carbohydrate parameters (i.e. carbohydrate intake, GI, fiber) in the models might blunt independent effects of GI and fiber on health outcomes.

### Strength and Limitation

This was the first study that found bilirubin could be affected by dietary factors. However, the mediating role of bilirubin levels on carbohydrate/GL-GDM associations contained uncertainty. The causal inference of the relationship remains controversial and needs to be confirmed by further dietary therapy on bilirubin levels and GDM prevention. Therefore, this secondary data analysis has little clinical implication on dietary recommendation. Instead, it sheds a new light into the underlying etiology of GDM, as well as the value of increasing serum bilirubin as an early protector of GDM following dietary modification, which called for future attention. The positive association between bilirubin and total carbohydrate intake (%E), may be explained by potential anti-inflammatory and anti-oxidative capacities, and regarding this, we still need more clinical trials to prove this benefit in at-risk population.

Some limitations of our study merit discussion. It is known that we were doing a secondary data analysis using cohort from a RCT program. Dietary counseling approaches, though controlled in the models, did affect the GDM outcome to some extent. As the data collection was not designed for this study aim, the data had limited availability that was impossible to be retrieved after years. Although we were able to adjust for a wide variety of lifestyle maternal factors, residual confounding was possible since some covariates were eliminated from analysis. Other factors affecting GDM such as vitamin D deficiency, FFAs, central obesity and polycystic ovary syndrome were not available in this study. Secondly, our study only included overweight and obese gravidae, so the results might not be generalized to normal-weight gravidae. It is noteworthy that insulin resistance and elevated triglyceride can be present in normal-weight people, especially when visceral adiposity exists, and this could not be fully explained by BMI (50). Hence, whether our findings are supportive to this population still needs investigation, preferably covering repeated insulin tolerance tests, visceral adiposity and lipid profile into analysis. Thirdly, the dietary intakes contained some error. Within-individual diet variability was not considered due to inability to repeat measurements on 24-h dietary recall. Besides, GI values were unavailable in some food items and insufficient calculation was possible. Future high-quality dietary data is needed to address this main flaw, as well as take more dietary factors (e.g., macronutrient subtypes, micronutrients, different vitamin supplement and biochemical indicators of dietary intake) into consideration. Self-reported information was also subject to underreporting and interviewer bias. While carbohydrate parameters seemed exchangeable across most maternal characteristics and results were robust in sensitivity analysis, the causal inference of examined associations was in uncertainty based on the observational nature of the study. Finally, due to limited statistical power in this small study, larger-scale, and well-designed investigations are warranted to draw firm conclusions.

## Conclusions

In summary, results from our study suggested mildly elevated serum bilirubin levels appeared to be in response to higher energies consumed from total carbohydrate intake during early pregnancy in overweight and obese gravidae. However, the mediating effect of bilirubin levels seemed modest, compared to direct effect of carbohydrate on GDM. Further large and well-designed investigations might help increase certainty, and give clues to the pathogenesis of GDM. The value of increasing serum bilirubin as an early protector of GDM following dietary modification, is also worthy of future attention.

## Data Availability Statement

The original contributions presented in the study are included in the article/Supplementary Materials, further inquiries can be directed to the corresponding author/s.

## Ethics Statement

The studies involving human participants were reviewed and approved by ethical review and approval were waived for this study, due to prior ethical clearance from the Ethics Committee of Children's Hospital of Fudan University (Approval No. 071-2012). The patients/participants provided their written informed consent to participate in this study.

## Author Contributions

WH and WY contributed to conception and design of the study. LW took charge of data collection and curation. WH performed the statistical analysis and wrote the original draft of the manuscript. WH, YZ, and XC performed software and visualization. WY provided supervision on the whole research procedure. All authors contributed to manuscript revision, read, and approved the submitted version.

## Conflict of Interest

The authors declare that the research was conducted in the absence of any commercial or financial relationships that could be construed as a potential conflict of interest.

## Publisher's Note

All claims expressed in this article are solely those of the authors and do not necessarily represent those of their affiliated organizations, or those of the publisher, the editors and the reviewers. Any product that may be evaluated in this article, or claim that may be made by its manufacturer, is not guaranteed or endorsed by the publisher.
