# Supplementary material for: Higher Serum Bilirubin Levels in Response to Higher Carbohydrate Intake During Early Pregnancy and Lower Gestational Diabetes Mellitus Occurrence in Overweight and Obese Gravidae
Source: Front Nutr. 2021 Aug 30;8:701422. doi: 10.3389/fnut.2021.701422 (PMC8435579; doi:10.3389/fnut.2021.701422)
Supplement: Supplementary file 1 [file Table_1.docx]

Supplementary Material

**Table S1.** Intercorrelations in relative intakes of dietary parameters.

| Correlation coefficients (r) | Total energy (kcal) | Carbohydrate  (%E) | Fat  (%E) | Protein  (%E) | Fiber  (g/1000kcal) | GI | GL (g/1000kcal) |
| --- | --- | --- | --- | --- | --- | --- | --- |
| Total energy(kcal) | 1 |  |  |  |  |  |  |
| Carbohydrate(%E) | 0.02 | 1 |  |  |  |  |  |
| Fat(%E) | 0.04 | -0.93 | 1 |  |  |  |  |
| Protein(%E) | -0.18 | -0.47 | 0.11 | 1 |  |  |  |
| Fiber(g/1000kcal) | 0.01 | 0.10 | -0.15 | 0.10 | 1 |  |  |
| GI | -0.02 | 0.18 | -0.14 | -0.14 | -0.28 | 1 |  |
| GL(g/1000kcal) | 0.001 | 0.86 | -0.79 | -0.43 | -0.05 | 0.61 | 1 |

Abbreviations: %E: percentage of total energy intake; GI, glycemic index; GL, glycemic load.

| **Table S2.** Baseline metabolic profiles in non-GDM and GDM participants (n=260)^1^ | | | |
| --- | --- | --- | --- |
|  | **Non-GDM**  **(n=203)** | **GDM**  **(n=57)** | **P-value** |
| BMI (kg/m^2^) | 28.34(3.09) | 28.99(2.69) | 0.146 |
| Gestational weight gain* (kg) | 5.95(4.10) | 5.48(3.56) | 0.445 |
| Fasting plasma glucose (mmol/L) | 4.63(0.43) | 4.78(0.43) | 0.019 |
| Insulin^2^ (μU/mL) | 8.4(5.9,11.9) | 10.7(7.9,12.5) | 0.002 |
| HbA_lc_ (%) | 5.26(0.73) | 5.42(0.29) | 0.113 |
| HOMA-IR^2^ | 1.69(1.16,2.57) | 2.15(1.65,2.89) | 0.002 |
| ALT^2^ (U/L) | 19(12,30) | 20(13,27) | 0.469 |
| TG^2^ (mmol/L) | 1.39(1.15,1.80) | 1.91(1.11,2.29) | 0.014 |
| TC^2^ (mmol/L) | 4.69(0.71) | 4.91(0.82) | 0.051 |
| HDL-C^3^ (mmol/L) | 1.49(0.29) | 1.51(0.35) | 0.822 |
| LDL-C^3^ (mmol/L) | 2.88(0.85) | 3.33(0.94) | 0.051 |
| SBP (mmHg) | 121.14(13.91) | 122.88(9.85) | 0.379 |
| DBP (mmHg) | 74.23(11.62) | 75.26(9.07) | 0.534 |
| Gestational hypertension | 39(19.50%) | 14(24.56%) | 0.405 |

^1^The distribution was summarized as mean (standard deviation) or frequency(%) if not specified.

^2^ Median (25th,75th). *From baseline till 24 gestational weeks.

^3^ Data available in 94/260 participants.

Abbreviations: BMI, body mass index; HbA1c, glycosylated hemoglobin; HOMA-IR, homeostasis model assessment of insulin resistance; ALT, alanine aminotransferase; TG, triglyceride; TC, total cholesterol; HDL-C, high-density lipoprotein cholesterol; LDL-C, low-density lipoprotein cholesterol; SBP, systolic blood pressure; DBP, diastolic blood pressure.

| **Table S3.** Associations of carbohydrate parameters with serum bilirubin levels in participants in sensitivity analysis ^1^ | | | | | |
| --- | --- | --- | --- | --- | --- |
|  | **TB(mmol/L)** | | **DB(mmol/L)** | | |
|  | **Adjusted regression coefficients (95%CI)** | **P** | | **Adjusted regression coefficients (95%CI)** | **P** |
|  | **n=259** |  | | **n=260** |  |
| **Total carbohydrate intake** |  |  | |  |  |
| change per 1%E increase in total carbohydrate intake | 0.026(-0.001,0.054) | 0.06 | | **0.014(0.002,0.026)*** | 0.01 |
| Tertile 1 | **Reference** | | | | |
| Tertile 2 | 0.828 (-0.033,1.688) | 0.05 | | 0.319(-0.044,0.682) | 0.08 |
| Tertile 3 | **0.870(0.011,1.730)*** | 0.04 | | **0.562(0.199,0.924)*** | 0.003 |
| **Fiber intake** |  |  | |  |  |
| change per 1g/1000kcal increase in fiber intake | -0.023(-0.114,0.067) | 0.61 | | -0.016(-0.055,0.022) | 0.39 |
| Tertile 1 | **Reference** | | | | |
| Tertile 2 | 0.017(-0.845,0.879) | 0.97 | | -0.222(-0.586,0.143) | 0.23 |
| Tertile 3 | 0.067(-0.832,0.967) | 0.88 | | -0.076(-0.457,0.304) | 0.69 |
| **Dietary Glycemic index** |  |  | |  |  |
| change per 1 increase  in dietary GI | 0.026(-0.013,0.064) | 0.18 | | -0.002(-0.018,0.015) | 0.85 |
| Low (≤55) | **Reference** | | | | |
| Medium (55-70) | -0.431(-1.603,0.740) | 0.46 | | -0.327(-0.824,0.171) | 0.19 |
| High (≥70) | 0.642(-0.681,1.966) | 0.34 | | -0.007(-0.570,0.555) | 0.97 |
| **Dietary Glycemic load** |  |  | |  |  |
| change per 1g/1000kcal increase in dietary GL | **0.019(0.006,0.032)*** | 0.005 | | **0.006(0.001,0.012)*** | 0.02 |
| Tertile 1 | **Reference** | | | | |
| Tertile 2 | 0.631(-0.206,1.469) | 0.20 | | 0.088(-0.270,0.446) | 0.63 |
| Tertile 3 | **1.071(0.237,1.905)*** | 0.01 | | **0.394(0.041,0.747)*** | 0.02 |

^1^adjusted for pregnancy alcohol drinking, baseline BMI, gestational week, maternal age, total energy intake, dietary counselling, hospital, baseline fasting glucose and triglyceride levels, parity, folic acid and vitamin supplement. Adjustment within carbohydrate parameters: carbohydrate: fiber and GI, fiber: carbohydrate and GI; GI: carbohydrate and fiber.

*Estimates with 95%CI beyond null.

Abbreviations: %E, percentage of total energy, TB, total bilirubin, DB, direct bilirubin, CI, confidence interval.

| **Table S4.** Associations of carbohydrate parameters with GDM in sensitivity analysis ^1^ | | |
| --- | --- | --- |
| **GDM: 57/260 (21.9%)** | **Adjusted odds ratio (95%CI)**  **n=260** | **P** |
| **Total carbohydrate intake** | | |
| 1%E | **0.97(0.94,0.99)*** | 0.02 |
| Tertile 1 | **Reference** |  |
| Tertile 2 | 0.89(0.40,1.97) | 0.78 |
| Tertile 3 | **0.39(0.15,0.97)*** | 0.04 |
| **Fiber intake** | | |
| 1g/1000kcal | 1.02(0.93,1.13) | 0.61 |
| Tertile 1 | **Reference** |  |
| Tertile 2 | 0.63(0.27,1.50) | 0.30 |
| Tertile 3 | 1.24(0.51,2.99) | 0.62 |
| **Dietary Glycemic index** | | |
| 1 unit | 1.00(0.96,1.04) | 0.98 |
| Low (≤55) | **Reference** |  |
| Medium (55-70) | 2.41(0.59,9.72) | 0.21 |
| High (≥70) | 1.35(0.28,6.62) | 0.70 |
| **Dietary Glycemic load** | | |
| 1g/1000kcal | **0.98(0.97,0.99)*** | 0.03 |
| Tertile 1 | **Reference** |  |
| Tertile 2 | 0.87(0.39,1.89) | 0.72 |
| Tertile 3 | **0.32(0.13,0.78)*** | 0.01 |

^1^adjusted for pregnancy alcohol drinking, baseline BMI, gestational week, maternal age, total energy intake, dietary counselling, hospital, baseline fasting glucose and triglyceride levels, parity, family history of diabetes or hypertension, folic acid and vitamin supplement, gestational hypertension, gestational weight gain. Adjustment within carbohydrate parameters: carbohydrate: fiber and GI, fiber: carbohydrate and GI; GI: carbohydrate and fiber.

*Estimates with 95%CI beyond null.

Abbreviations: %E, percentage of total energy, GDM, gestational diabetes mellitus, CI, confidence interval.
